# Phenotypic high-throughput screening platform identifies novel chemotypes for necroptosis inhibition

**DOI:** 10.1038/s41420-020-0240-0

**Published:** 2020-02-11

**Authors:** Hugo Brito, Vanda Marques, Marta B. Afonso, Dean G. Brown, Ulf Börjesson, Nidhal Selmi, David M. Smith, Ieuan O. Roberts, Martina Fitzek, Natália Aniceto, Rita C. Guedes, Rui Moreira, Cecília M. P. Rodrigues

**Affiliations:** 1 Research Institute for Medicines (iMed.ULisboa), Faculty of Pharmacy, Universidade de Lisboa, 1649-003 Lisbon, Portugal; 2 Hit Discovery, Discovery Sciences, R&D Biopharmaceuticals, AstraZeneca, Boston, MA 02451 USA; 3 Hit Discovery, Discovery Sciences, R&D Biopharmaceuticals, AstraZeneca, Gothenburg, 431 83 Sweden; 4 Emerging Innovations Unit, Discovery Sciences, R&D, AstraZeneca, Cambridge, CB4 0WG UK; 5 Hit Discovery, Discovery Sciences, R&D Biopharmaceuticals, AstraZeneca, Cambridge, CB4 0WG UK; 6 Hit Discovery, Discovery Sciences, R&D Biopharmaceuticals, AstraZeneca, Alderley Park, Macclesfield, SK10 4TG UK

**Keywords:** Screening, Drug development

## Abstract

Regulated necrosis or necroptosis, mediated by receptor-interacting kinase 1 (RIPK1), RIPK3 and pseudokinase mixed lineage kinase domain-like protein (MLKL), contributes to the pathogenesis of inflammatory, infectious and degenerative diseases. Recently identified necroptosis inhibitors display moderate specificity, suboptimal pharmacokinetics, off-target effects and toxicity, preventing these molecules from reaching the clinic. Here, we developed a cell-based high-throughput screening (HTS) cascade for the identification of small-molecule inhibitors of necroptosis. From the initial library of over 250,000 compounds, the primary screening phase identified 356 compounds that strongly inhibited TNF-α-induced necroptosis, but not apoptosis, in human and murine cell systems, with EC_50_ < 6.7 μM. From these, 251 compounds were tested for RIPK1 and/or RIPK3 kinase inhibitory activity; some were active and several have novel mechanisms of action. Based on specific chemical descriptors, 110 compounds proceeded into the secondary screening cascade, which then identified seven compounds with maximum ability to reduce MLKL activation, IC_50_ >100 μM, EC_50_ 2.5–11.5 μM under long-term necroptosis execution in murine fibroblast L929 cells, and full protection from ATP depletion and membrane leakage in human and murine cells. As a proof of concept, compound SN-6109, with binding mode to RIPK1 similar to that of necrostatin-1, confirmed RIPK1 inhibitory activity and appropriate pharmacokinetic properties. SN-6109 was further tested in mice, showing efficacy against TNF-α-induced systemic inflammatory response syndrome. In conclusion, a phenotypic-driven HTS cascade promptly identified robust necroptosis inhibitors with in vivo activity, currently undergoing further medicinal chemistry optimization. Notably, the novel hits highlight the opportunity to identify new molecular mechanisms of action in necroptosis.

## Introduction

Deregulation of apoptosis represents a key pathologic event in human disease, thus constituting an appealing therapeutic target^1^. Despite the benefits of blocking cell death, the scarcity of “druggable” targets in the apoptotic signalling pathway associated with safety concerns has hampered the use of apoptosis inhibitors in clinical practice^2^. Recent evidence has shown that necroptosis, a type of regulated necrosis, also plays a key pathogenic role in a vast range of human diseases^3^, while holding potential for clinical targeting^4^. At the molecular level, necroptosis can be triggered by DNA damage, immune receptors and viruses, or by death receptors of the tumour necrosis factor (TNF) superfamily, such as FasR, TRAILR 1/2 and TNFR1. Upon interaction with TNF-α, TNFR1 trimerizes and assembles with TRADD, RIPK1, CYLD, TRAF2 and cIAP 1/2, thus forming the membrane-associated protein complex I. When signalling complexes NEMO/IKKα/IKKβ, TAB1/2/3/TAK1 and LUBAC are further recruited and activated, the NF-κB and MAPK survival pathways are activated. Alternatively, receptor-interacting kinase 1 (RIPK1), FADD and caspase-8 form cytosolic complex IIa to activate the caspase cascade and induce apoptosis. When caspase-8 activity and/or survival signalling are inhibited, RIPK1/FADD interacts with RIPK3 and mixed lineage kinase domain-like protein (MLKL) to form the necrosome, or complex IIb. Mechanistically, phosphorylated MLKL triggers cell membrane permeabilization and consequent release of pro-inflammatory intracellular contents to the extracellular space^5^.

The study of necroptosis in RIPK1/3 or MLKL knockout models of disease or in animals treated with pharmacologic inhibitors, such as necrostatin-1 (Nec-1), have identified necroptosis as a key pathway participating in a plethora of disorders, such as ischaemia-reperfusion injury^6^, neurodegeneration^7^, atherosclerosis^8^, ethanol-induced liver injury^9^, acute pancreatitis^10^ and cholestasis^11^, among others. The first necroptosis inhibitor, Nec-1, was identified through a phenotypic high-throughput screening (HTS) for chemical inhibitors of necrotic cell death induced by TNF-α and Z-VAD-FMK in human monocytic U937 cells^12^. However, Nec-1, which was shown to inhibit RIPK1^13^, is modestly potent and often associated with off-target effects. These suboptimal properties, shared by other necrostatins, have led to efforts to identify the next generation of necroptosis inhibitors. Nevertheless, phenotypic^14^ or target-based^15^ screen of diverse chemical libraries, yielded necroptosis inhibitors with poor specificity, off-target effects, limited bioavailability and absence of inter-species cross-activity. This, coupled with necroptosis-independent function of RIPKs and MLKL, has prevented these compounds from reaching further pre-clinical development^16^.

In this study, we developed a phenotypic-driven HTS strategy to find novel necroptosis inhibitors using a high-quality library of 251,328 compounds. The three-stage screening workflow included *in silico*, in vitro and in vivo assays, yielding novel hits that represent an opportunity to identify new molecular mechanisms of action in necroptosis.

## Results

### Three-step HTS cell-based phenotypic cascade for hit selection

To identify novel necroptosis inhibitors from a diverse compound library (AstraZeneca; Cambridge, UK), we designed and validated a three-step primary cell-based HTS phenotypic assay, established on a 384-well microtiter platform, taking into account assay reproducibility, sensitivity and costs^17^ (Fig. S1). Pilot tests were performed to fine-tune in vitro assay methodologies, including cell density, incubation times (pre- or co-incubation of TNF-α and Nec-1), endpoint phenotypic assays (total ATP or adenylate kinase (AK) enzyme release), and plate layout (data not shown). Overall, lower cell density and 8 h incubation periods increased signal response after stimulation with TNF-α. For reproducibility, positive and negative controls were positioned in the middle of the plate to avoid major temperature imbalances or excessive evaporation^18^. AK enzyme release compared with total ATP levels exhibited higher sensitivity in reflecting TNF-α-induced cell death, functioning as a reporter of cell lysis and hence, necroptosis^19^.

**Fig. 1:**
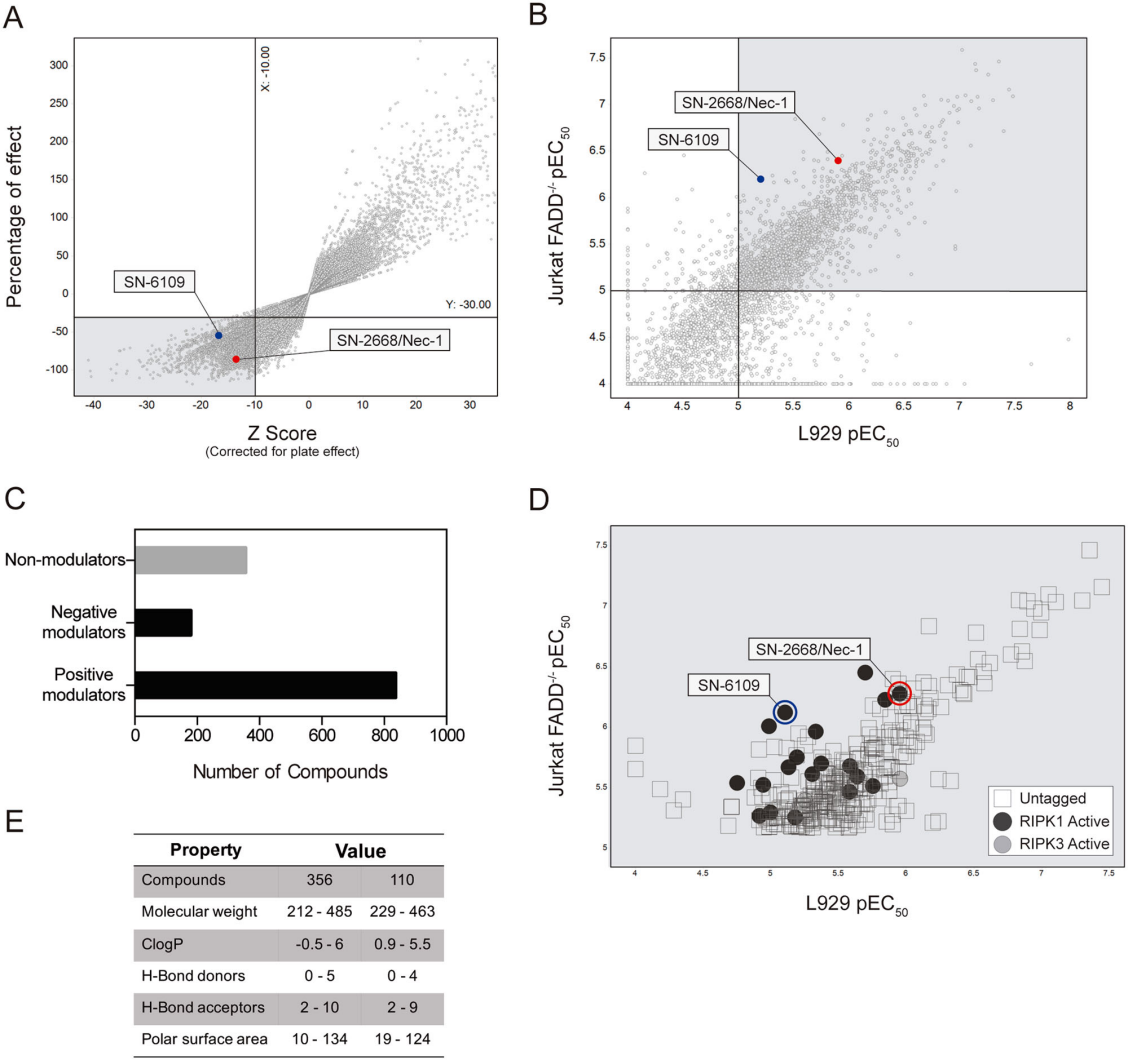
A phenotypic cell-based compound screen identifies 356 necroptosis inhibitors from a 251,328 compound library. Proof of concept compound SN-6109 and SN-2668/Nec-1 are highlighted. **a** Evaluation of compound ability to prevent necroptosis execution. Membrane integrity values are depicted on *Y* axis as percentage of control (DMSO = 0; no addition = −100; Nec-1 at 29.2 μM > −100) for compounds tested at a single dose of 31.7 μM in murine L929 cells exposed to 10 ng/mL mTNF-α for 8 h. **b** Correlation of compound half maximal effective concentration (EC_50_), determined in murine L929 and human Jurkat FADD^-/-^ cells in a 10-point dose-response concentration (0.004 to 100 μM). **c** Apoptosis modulation activity of tested compounds evaluated in human Jurkat E6.1 cells incubated with 0.5 μg/mL CHX and test compounds at a 4-point dose-response (0.03 to 30 μM) for 8 h. Cell viability data represent a single experiment normalized to untreated control. Cell viability was assessed using a luminescence-based readout for AK release and Caspase-Glo 3/7. **d** Evaluation of tested compounds for RIPK1 and RIPK3 kinase inhibitory activity at 1 μM using radiometric-binding and FRET-based assays, respectively. **e** Drug-like physicochemical properties for the 356 hits and 110 selected compounds.

A total of 251,328 small-molecule compounds were screened in L929 cells incubated with mTNF-α alone or together with test compounds at 31.7 μM for 8 h. Intraplate controls for data normalization consisted of untreated cell wells. L929 cells co-incubated with mTNF-α and SN-2668/Nec-1 served as positive controls (Fig. S2A). For hit selection, exclusion criteria included both qualitative and quantitative parameters, namely Z Score of −10 and percentage of effect of −30% (necroptosis inhibition superior to 30%). In HTS step one (primary screening), valid data was obtained for 247,738 compounds, 3353 of which were within the established hit selection criteria, corresponding to a 1.4% hit rate for the full library (Fig. 1a). After the initial screen, multiple rounds of near neighbour searching based both on chemical fingerprint distances to the initial seed structure as well as substructure searches, within the complete collection of over 2 million compounds (AstraZeneca), resulted in further augmentation of the original hit list, as well as elucidation of new structure activity relationships.

The second screening step aimed to determine the EC_50_ of selected compounds. L929 and Jurkat FADD^-/-^ cells were co-incubated with TNF-α and test compounds at 10-point concentration range (0.004–100 μM) for 8 h. Compound-mediated inhibition of necroptosis was assessed through AK release and the EC_50_ calculated. Jurkat cells were used to validate results in human cells. Hit compounds presenting a pEC_50_ > 5 in both cell lines were selected for step three of the HTS workflow (Fig. S1). In HTS step two, 4374 compounds (3353 hits from HTS step one plus 1021 near neighbours) were evaluated for potency by dose-response curves. From those, 1,438 hit compounds passed the selection criteria on both cell lines, corresponding to a 31.7% hit rate. In contrast, only 1110 and 483 compounds displayed necroptosis inhibitory activity in L929 or Jurkat FADD^-/-^ cells, respectively (Fig. 1b).

Since apoptosis and necroptosis share key molecular players^20^ and some RIPK3 necroptosis inhibitors may induce caspase-dependent cytotoxicity^21^, hit compounds were tested for modulation of apoptosis activity in step three of the HTS workflow. Preliminary tests prior to screening encompassed the evaluation of several apoptosis inducers, such as FasL, doxorubicin, cycloheximide (CHX) and staurosporine, for their capability to modulate caspase-3/-7 activity in human Jurkat E6.1 T-cells, using Z-VAD-FMK and SN-2668/Nec-1 as negative and positive controls, respectively^22^. CHX was chosen for downstream assays due to its consistency between tests and because CHX alone increased caspase-3/-7 activity without compromising cell membrane integrity. As expected, SN-2668/Nec-1 failed to modulate caspase activity (Fig. S2A). For the screening, Jurkat E6.1 T-cells were co-incubated with CHX and test compounds at 4-point concentration range (0.03–30 μM) for 8 h. Intraplate controls for data normalization consisted of untreated and CHX-only treated cells. Compounds able to modulate caspase-3/-7 activity were excluded from the screening with the remaining considered high-confidence hits and advancing for the next phase of the validation process (Fig. S1). In HTS step three, 356 compounds displayed non-interference with caspase activity in all four tested doses, corresponding to a 24.8% hit rate. On the other hand, 158 and 859 compounds positively and negatively modulated apoptosis activity, respectively, at least in one tested dose (Fig. 1c). Hits fit to 192 chemical clusters of which 124 were singletons, corresponding to 0.14% hit rate from the initial compound library. Valid data was achieved for 96.4% of the tested compounds from the initial library.

### RIPK1 and RIPK3 kinase activity by selected compounds

Most currently available necroptosis inhibitors, including necrostatin compound family^16^, ponatinib, pazopanib^23^, dabrafenib^24^, tozasertib^25^ and GSK inhibitors^15^, directly inhibit RIPK1 or/and RIPK3 kinase activities. Based on that, HTS step two hits plus singletons of interest comprising a total of 1485 compounds were screened for RIPK1 and RIPK3 kinase activity inhibitory potential using radiometric binding- and FRET-based assays, respectively. From this set, only 32 and 22 compounds inhibited RIPK1 or RIPK3 kinase activity, respectively, by more than 50% at 1 μM. A total of 25 compounds demonstrated IC_50_ < 1 μM for RIPK1 and 15 compounds with IC_50_ < 1 μM for RIPK3. From those, only one compound demonstrated an IC_50_ at both RIPK1 and RIPK3. Only 18 RIPK1 and 3 RIPK3 inhibitors were hits from step 3. Remarkably, 18 were not related to known scaffolds and represented novel chemotypes with the ability to inhibit RIPK1/3 activity. The remaining 338 compounds from the HTS triage were inactive in both kinase assays, thus leaving open the possibility to find novel molecular mechanisms of action (MMoA) (Fig. 1d).

### Selection of compounds for further validation

Selected compounds fulfilled the rule of five (Ro5) for physicochemical parameter ranges; no more than one violation of the following criteria: molecular weight (MW) <500, lipophilicity (logP) <5, H-bond donors (HBD) <5, H-bond acceptors (HBA) <10) and solubility indicators such as polar surface area (PSA), important for oral bioavailability^26^. Prioritization of clusters containing narrow physicochemical parameters, alongside selection on structural features and frequent hitting behaviours led to 110 compounds belonging to 28 clusters for further exploitation in a secondary screening phase, as well as for chemical synthesis of novel optimized derivatives (Fig. 1e).

### Long-term potency and toxicity of hits in a secondary counter-screen phase

**Fig. 2:**
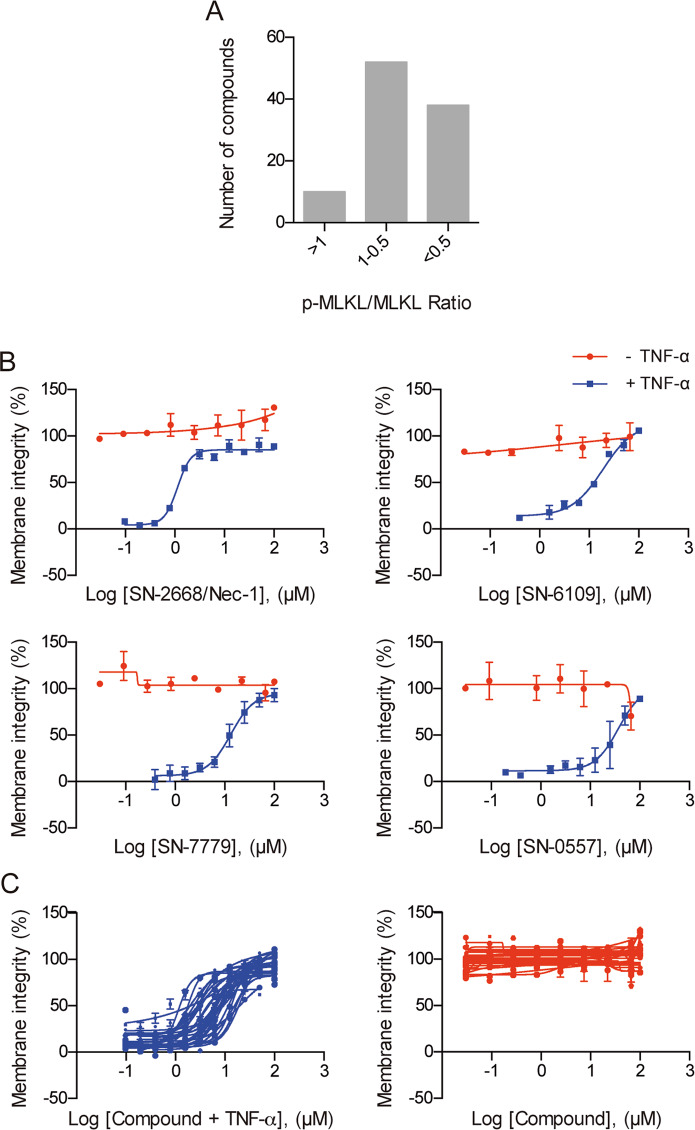
Secondary-stage cascade for hit validation and compound refinement for further studies. **a** Modulation of p-MLKL/MLKL ratio by tested compounds was evaluated in murine L929 cells exposed to 10 ng/mL mTNF-α and tested compounds at their EC_50_ for 8 h. Whole cell extracts were analysed by immunoblotting. Data shown are representative of one single experiment. **b**, **c** Selected compound half maximal effective concentration (EC_50_) preventing necroptosis execution and half maximal inhibitory concentration (IC_50_) in murine L929 cells in the presence or absence of 10 ng/mL mTNF-α and compounds on 10-point dose-response concentration (0.01–100 μM) for 24 h. Data represent mean values ± SEM of three independent experiments normalized to untreated control. Cell viability was assessed using a colorimetric-based readout for LDH release.

The ability of compounds to interfere with specific steps of the necroptosis pathway, as well as inherent cytotoxicity and long-term necroptosis inhibitory effects were evaluated using in vitro necroptosis models (Fig. S1). Firstly, the 110 compounds were tested for their ability to modulate TNF-α-induced MLKL phosphorylation^27^, a key necroptotic effector downstream of RIPK3^13^. Results showed that 100 compounds were capable of decreasing p-MLKL/MLKL ratio, while 10 compounds had either little or the opposite effect (Fig. 2a and Fig. S2B). Second, for evaluation of long-term necroptosis protection EC_50_, L929 cells were co-incubated with mTNF-α and tested compounds on a 4-point concentration range (1–30 μM) for 24 and 48 h. Membrane integrity was evaluated by both AK and LDH release to exclude potential false positives^28^. Only seven compounds showed significant toxicity at the highest dose, 28 were inactive at least in one of the tested cytotoxicity assays, while 75 compounds completely prevented necroptosis in at least one of the tested dosages (data not shown). Taken together the ability to significantly reduce p-MLKL levels, lack of toxicity and ability to inhibit necroptosis similarly to SN-2668/Nec-1, 27 compounds were selected for further in vitro testing, where both EC_50_ and IC_50_ were determined in L929 cells (in the absence or presence of TNF-α) on a 10-point concentration range (0.01–100 μM) for 24 h. Compounds showed no toxicity (IC_50_ > 100 μM) with EC_50_ ranging from 0.6 to 30.0 μM (Fig. 2b, c).

**Fig. 3:**
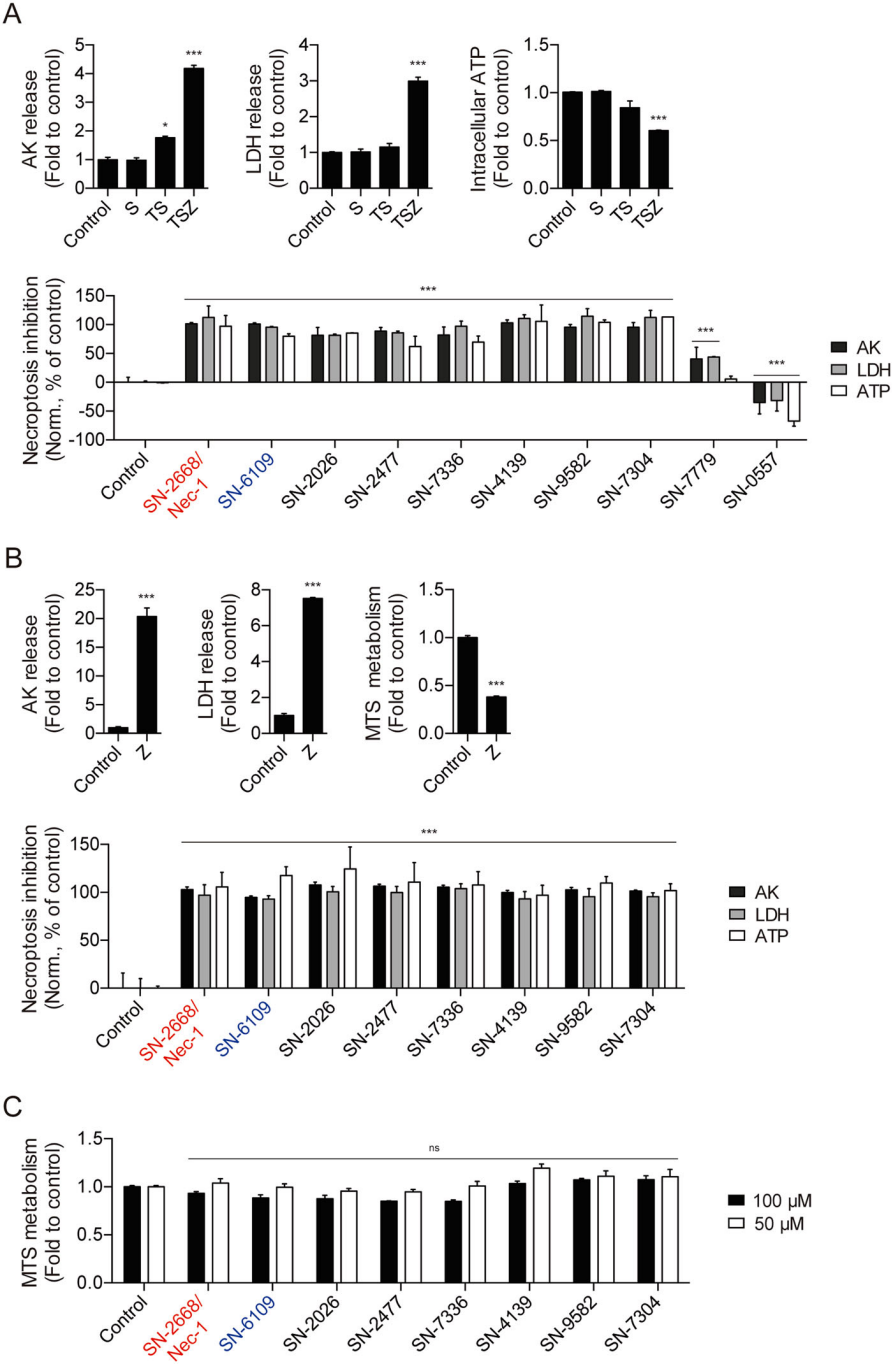
Secondary-stage cascade for hit validation and compound refinement for further studies. **a** Compound ability to prevent necroptosis execution in HT29 cells co-incubated with 20 ng/mL hTNF-α (T) together with 250 nM Smac mimetic BV6 (S) and 10.5 μM Z-VAD-FMK (Z), and selected compounds at 10 μM for 24 h. **b** Selected compound ability to prevent necroptosis execution in BV2 cells co-incubated with 25 μM Z-VAD-FMK (Z) and compounds at 10 μM for 24 h. **c** Selected compound toxicity evaluation in HepG2 cells incubated with test compounds at 100 and 50 μM for 24 h. Data represent mean values ± SEM of three independent experiments normalized to untreated control. Cell viability was assessed using a luminescence-based readout for AK release and ATP production, and a colorimetric-based readout for LDH release and MTS metabolism. ns, not significant; *p < 0.05, ***p < 0.001.

Necroptosis inhibition properties were further examined using two additional cellular models of regulated necrosis. First, in human adenocarcinoma HT29 cells, and then in immortalized murine microglial BV2 cells, where we co-incubated a mixture of human or murine TNF-α, Smac mimetic birinapant, pan-caspase inhibitor Z-VAD-FMK^29^ or Z-VAD-FMK alone^30^, and tested compounds at 10 μM for 24 h. From the 27 tested compounds, seven compounds plus SN-2668/Nec-1 completely rescued membrane integrity and ensured metabolic cell viability, evaluated by AK and LDH release and by ATP intracellular levels, respectively. From the remaining compounds, 18 displayed protections similar or better than SN-7779, and curiously the SN-0557 compound potentiated membrane leakage in this cellular model (Fig. 3a). Subsequently, the seven compounds plus SN-2668/Nec-1 tested on BV2 cells plus Z-VAD-FMK displayed complete protection against necroptosis execution (Fig. 3b). In parallel, preliminary data for compound liver toxicity was extrapolated using HepG2 cells. Tested compounds did not decrease cell viability at 100 and 50 μM for 24 h (Fig. 3c).

**Fig. 4:**
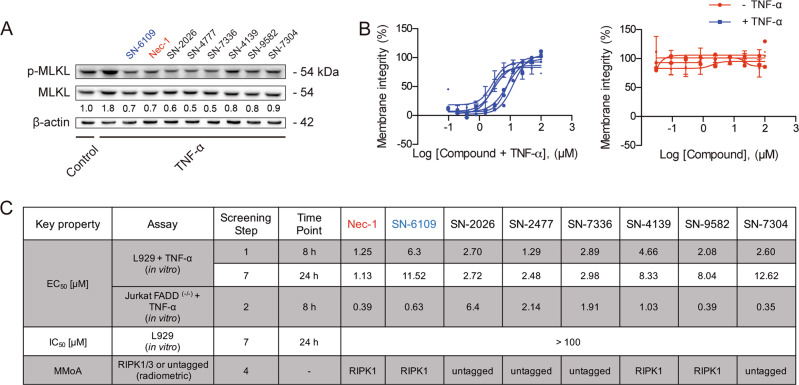
In vitro assays for the most interesting compounds. **a** Modulation of p-MLKL/MLKL ratio was evaluated in murine L929 cells exposed to 10 ng/mL mTNF-α and tested compounds at their EC_50_ for 8 h. Whole cell extracts were analysed by immunoblotting. Data shown are representative blots and quantification of p-MLKL/MLKL ratio from one single experiment, in fold-change from control. Blots were normalized to endogenous β-actin. **b** Selected compound half maximal effective concentration (EC_50_) preventing necroptosis execution (left) and half maximal inhibitory concentration (IC_50_) in murine L929 cells in the presence or absence of 10 ng/mL mTNF-α and compounds on 4-point dose-response concentration (0.01–100 μM) for 24 h (right). Data represent mean values ± SEM of three independent experiments normalized to untreated control. Cell viability was assessed using a colorimetric-based readout for LDH release. **c** In vitro screening results for seven selected compounds at different stages of the HTS cascade.

Selected compounds decreased p-MLKL/MLKL similarly to Nec-1 (Fig. 4a). In addition, the EC_50_ was comparable to that of Nec-1 in L929 cells for 24 h of incubation, without any relevant toxicity (Fig. 4b, c). Three compounds inhibited RIPK1 kinase activity and the remaining displayed unknown mechanism (Fig. 4c). Of note, the unknown MMoA compounds belong to six distinct chemical clusters, currently being explored for further chemical expansion based on physicochemical properties. With these results in hand, we then analysed the binding mode of SN-6109 in RIPK1 and evaluated its in vivo efficacy.

### Binding mode of SN-6109 in RIPK1

**Fig. 5:**
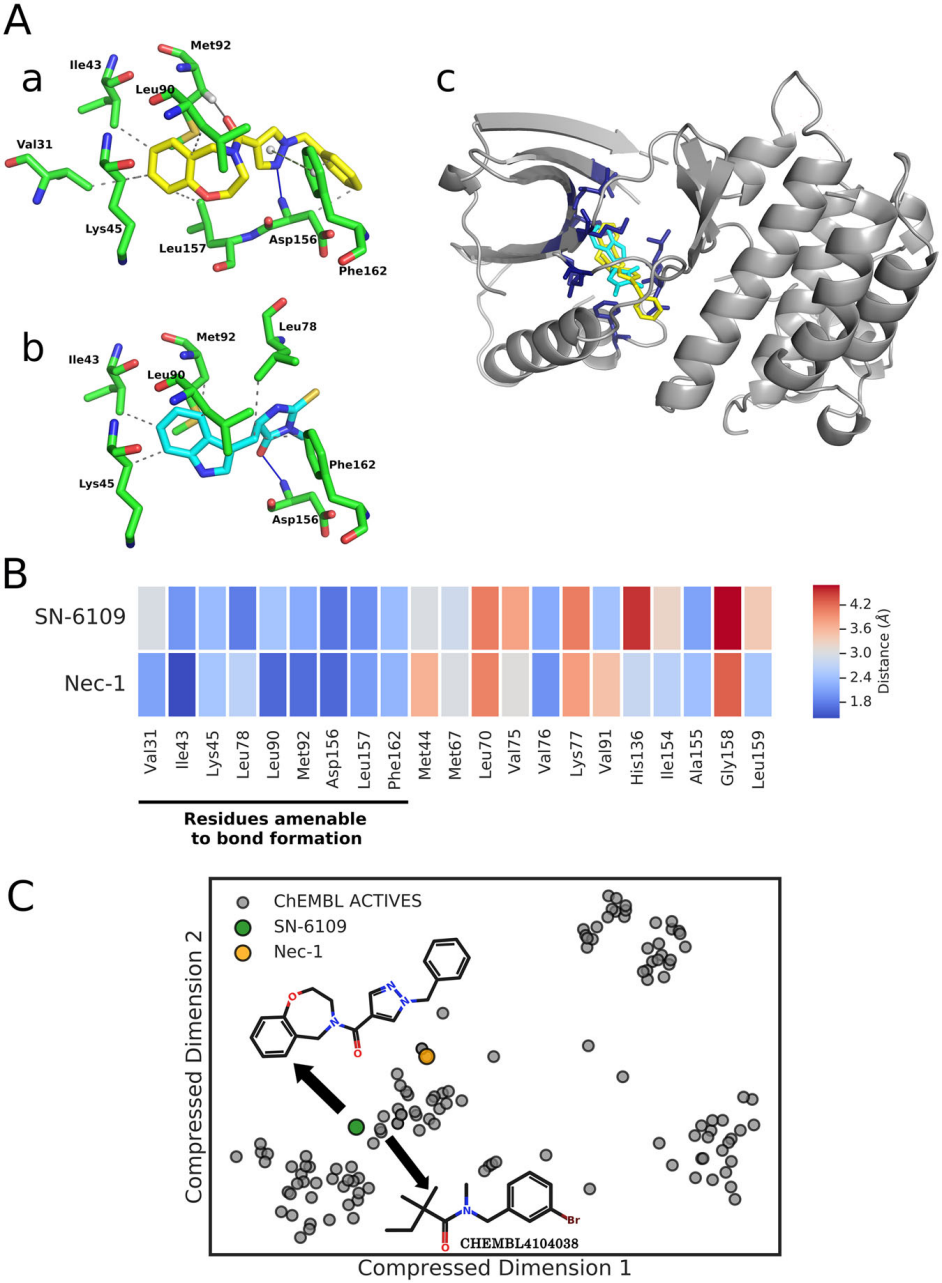
Structural analysis and visualization of SN-6109 and Nec-1 docked poses inside RIPK1 binding pocket. **a** Highest scored docking pose of SN-6109 inside RIPK1 binding site; key interacting residues are shown as sticks (a). Highest score docking pose of Nec-1 inside RIPK1 binding site; key interacting residues in the vicinity are shown as sticks (b). Superimposed docking poses of SN-6109 and Nec-1 in complex into RIPK1 binding site; receptor structure shown as ribbons (c). **b** Heat map of amino acid residues within 5 Å of SN-6109 and Nec-1, either able to establish an interaction with the ligand or not. **c** Comparison of chemical space coverage between ChEMBL actives and SN-6109. The scatter plot was produced by t-SNE multidimensional scaling from Morgan fingerprints (folded over 1024 bits), with SN-6109 and Nec-1 highlighted. The common scaffold between SN-6109 and its closest ChEMBL neighbour is also highlighted and was derived by maximum common substructure. The protocol for selection of the pdb file used for the docking studies as well as protein and ligand preparation is included in Supporting Information.

Molecular docking experiments were carried out with available RIPK1 protein structures to investigate and characterize the binding pose and key interactions of proof of concept SN-6109 with the active site residues (Fig. 5a). Among the RIPK1 X-ray structures available and tested in preliminary studies, 5HX6 (2.23 Å) was the only structure that accommodated both large and small ligands and, therefore, was used in further studies. Ligand interaction profiles were determined using PLIP^31^ and MOE 2019.01. The results revealed that SN-6109 is placed inside the kinase-binding pocket, interacting mainly with Val31, Ile43, Lys45, Leu90, Met92, Asp156, Leu157 and Phe162. The proposed binding mode for SN-6109 is somewhat similar to that of Nec-1, with docking score values of 79.7 and 65.2, respectively. In addition, SN-6109 and Nec-1 share very similar residue profiles (Fig. 5b). The interaction profile suggests an allosteric binding mode. Importantly, SN-6109 is not covered by the chemical space of RIPK1 inhibitors, with the closest neighbour in ChEMBL database sharing only a small scaffold (Fig. 5c).

### In vivo efficacy of SN-6109 in TNF-α-induced SIRS in mice

**Fig. 6:**
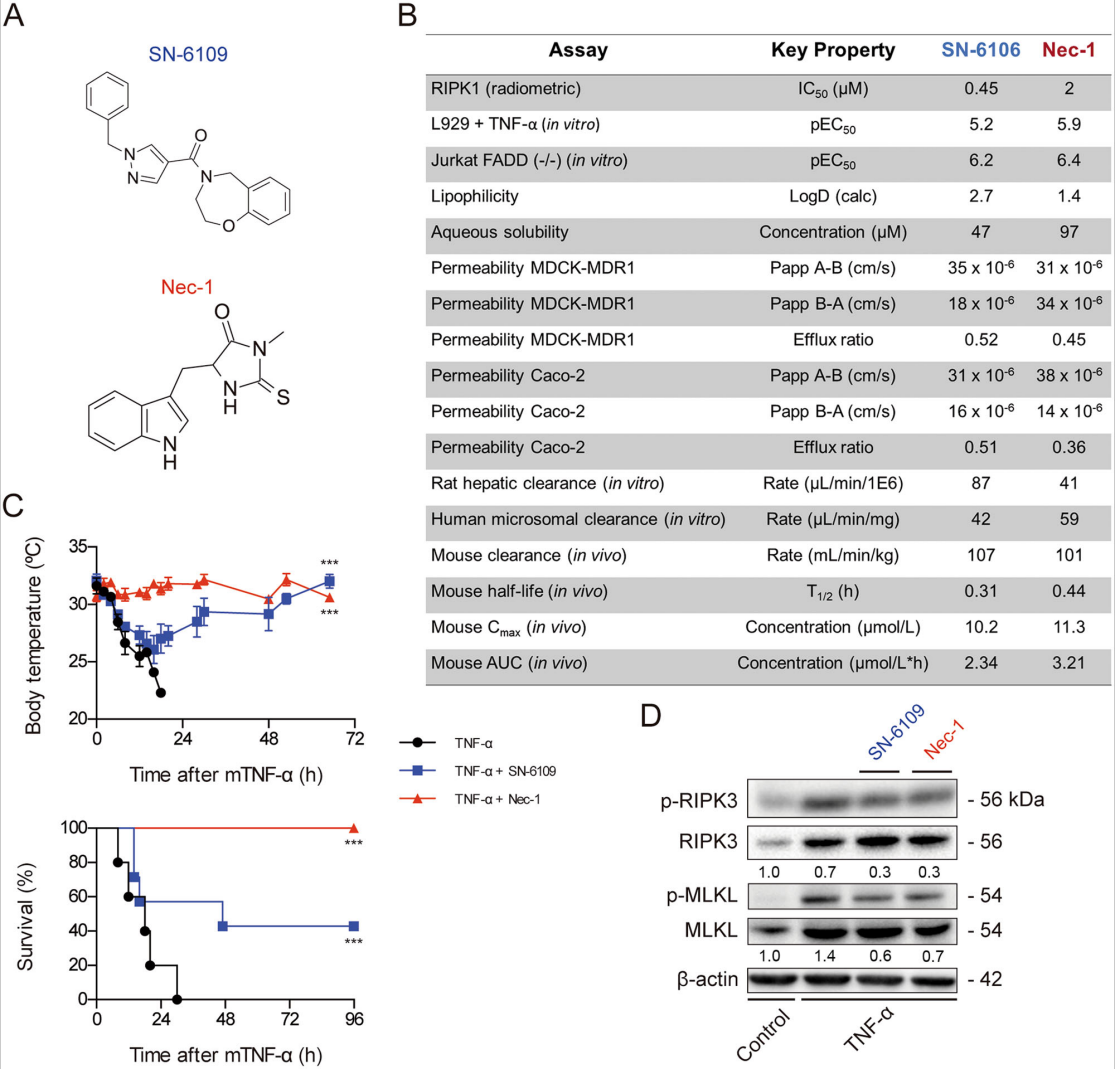
SN-6109 efficacy against TNF-α-induced SIRS in mice. SN-6109 and Nec-1 were injected i.v. at a dose of 5 mg/kg body weight, before the challenge with mTNF 0.38 mg/kg. **a** SN-6109 and Nec-1 chemical structure. **b** SN-6109 and Nec-1 RIPK1 kinase modulation, pharmacodynamic and pharmacokinetic properties. **c** TNF-α shock-associated hypothermia and survival curves. **d** Expression and activity of key necroptosis proteins in liver tissue. Representative blots and quantification of p-RIPK3/RIPK3 and p-MLKL/MLKL ratios in fold-change from control. Blots were normalized to endogenous β-actin. Data represent mean values ± SEM of six to seven animals in each group. ***p < 0.001.

To confirm that the screening strategy leads to hits functionally active in vivo, we tested one of the hits for its ability to protect against RIPK-driven inflammation in TNF-α-induced systemic inflammatory response syndrome (SIRS). This mouse model of sterile sepsis^32^ is dependent on RIPK1 and RIPK3 kinase activity and independent of apoptosis and inflammasome activity^33^. SN-6109, a benzoxazepine amide was selected for its presumed MMoA similar to Nec-1 (Fig. 6a). Proof of concept SN-6109 showed direct inhibition of RIPK1 kinase activity (IC_50_: 0.45 μM in radiometric kinase assays), good physical chemical properties (aqueous solubility: 47 μM; low lipophilicity: 2.7), but moderate/high in vitro metabolic clearance (rat hepatic clearance: 87 μL/min/1E6; human microsomal clearance: 42 μL/min/mg). Good drug-like physiochemical permeability properties were predicted from MDCK-MDR1 and Caco-2 cell-based permeability assay data^34^. Finally, in vivo SN-6109 pharmacokinetics was not optimal, showing metabolic stability *t*_1/2_ of 0.31 h, *C*_max_ of 10.2 μmol/L and *T*_max_ of 0.03 h. Nec-1 displayed *t*_1/2_ of 0.44 h, *C*_max_ of 11.3 μmol/L and *T*_max_ of 0.03 h (Fig. 6b).

Using the SIRS mouse model, SN-6109 and Nec-1 were administered at same dose and time prior to mTNF-α injection. Single injection of mTNF-α resulted in 0% survival, as expected from the lethal dose^35^. Mice pre-treated with Nec-1 all survived and were fully protected from hypothermia, as previously reported^33^, whilst almost 50% of mice survived after treatment with SN-6109 and gradually recovered their basal temperature in 48 h post-injection (Fig. 6c). Moreover, SN-6109 and Nec-1 decreased p-MLKL/MLKL and p-RIPK3/RIPK3 ratios in mouse liver tissue compared with mTNF-α alone (Fig. 6d). These results show that SN-6109 showed efficacy in an in vivo model of tissue injury and inflammation driven by RIPK1/3-dependent cell death.

## Discussion

In this study, a phenotypic HTS workflow was designed and implemented to identify necroptosis inhibitors in a target-agnostic approach. The majority of selected hits act through either unknown targets or novel MMoA for well-known targets and are currently being further explored, thus potentially enabling mitigation of the incomplete understanding of necroptosis physiology in a disease perspective^36^.

The disease relevance of the assay system selected for cell-based phenotypic HTS was a *sine qua non* condition to generate meaningful data toward finding promising necroptosis inhibitors. By the use of multiple cell lines, from different species and tissues, such as L929^37^, Jurkat FADD^−/−^^38^, HT29^29^ and BV2 cells^30^, all well-established cellular models to study necroptosis^39^, we ensured the specificity and fitness for purpose of the selected hits as necroptosis inhibitors. The stimulus TNF-α leads to the activation of downstream pathways that assemble the necrosome, a cytosolic signalling platform, which activates the downstream effector MLKL^40^ in a RIPK1-(in)dependent and RIPK3-dependent manner^41^. Moreover, TNF-α is the central regulator of inflammation, frequently upregulated in necroptotic associated disorders, such as inflammatory and neurodegenerative diseases^42,43^, ischaemia-reperfusion^6^ and solid organ injury^44^. Finally, throughout the workflow, key markers of necroptosis were evaluated in vitro and in vivo; these included permeabilization of plasma membrane upon stimulation by external stimuli, evaluated by intracellular protein release-based assays^45^; depolarization of mitochondria and impairment of ATP synthesis during necroptosis, evaluated by NAD(P)H-dependent dehydrogenase enzymes activity and quantification of intracellular ATP levels, respectively^30^; absence of apoptosis, measured by caspase-3/-7 activity^46^; RIPK3 and MLKL phosphorylation, assessed by immunoblotting^47^; and induction of systemic inflammatory response in mice as a model of human sepsis and protection from death^48^.

The cascade was designed to increase the chances of getting new validated necroptosis inhibitors with potential for medicinal chemistry development towards pre-clinical in vivo experiments and future human medicinal use. Different factors were considered to validate the selected strategy and reinforce robustness, reliability, relevance of the results, as well as fitness for purpose. Firstly, the pharmacological relevance of the assay was ensured by the capacity to select hit compounds at the end of the two main stages by using validated necroptosis inhibitors. SN-2668/Nec-1, a necroptosis RIPK1-kinase inhibitor was used as a blind positive control throughout the screening procedure^13^. Furthermore, tested compounds were used on the primary screening at a concentration of 31.7 μM, which is above the typical 1–10 μM^49^, thus filtering new necroptosis inhibitor chemical scaffolds with potential to be further modified by medicinal chemistry, while excluding compounds with associated toxicity.

The execution of the three-step primary HTS workflow presented a 0.14% hit rate, similarly to other screens^23,50^. The limited success of typical single-step HTS was overcome here by performing two subsequent orthogonal assays, in a high-throughput scale^28^. Moreover, HTS step one selection criteria for hits comprised quantitative (percentage of effect < −30%) and qualitative (Z Score < −10) thresholds, thereby excluding false positives or skewed plate signal. This strategy allowed reducing from around 14,000 putative hits to 3516 hits with high confidence. HTS step two, where drug potency was evaluated by dose-response curves, estimated pharmacodynamic properties of drugs that underlie the basis of most molecular interactions in biological systems^51^. Furthermore, using both human and murine cell systems in parallel safeguarded interspecies activity of molecules crucial for future pre-clinical and clinical trials^52^. This strategy allowed excluding 1548 compounds with single activity in one of the tested cell lines. At HTS step three, 1017 compounds were excluded due to interference with caspase activity under apoptosis execution. This issue was contemplated as some necroptosis inhibitors may promote a switch to apoptosis^53^, opposite to the activation of necroptosis in the presence of caspase inhibitors^54^. This step excluded compounds that would interact with key necroptotic players in an apoptosis manner independent of pro-necroptotic activity^21^. Additionally, false positives by compound interference with the biochemical assay format and technique was mitigated by the use of two output reporters, AK release and caspase-3/-7 activity^55^. A limited number of molecules were removed due to common false positive hit rates and known bad actors based on prior HTS screens using a pBSF score, which is the negative log of binomial survivor function to quantify frequent hitters^56,57^. In parallel, 1485 compounds were tested in vitro for ability to modulate RIPK1 and RIPK3 kinase activity. This step allowed in a preliminary phase filtering some of the hits for the ability to interact with two common targets. From the tested library, 40 compounds (2.7%) displayed RIPK1 or/and RIPK3 kinase activity. Of these, 21 positively modulated kinase activity for RIPK1 and RIPK3 without interfering with apoptosis execution. Here, we describe for the first time the potential of 18 novel compounds with the ability to interact with RIPK1 or RIPK3 with measurable IC_50_ values < 1 μM. From those with unknown mechanism of action, the opportunity exists to uncover new molecular targets responsible for necroptosis execution. From the 356 hits, 110 compounds were selected for the secondary counter screen, based on physicochemical and biological characteristics, assuring potential for future lead optimization and clinical application^58^.

The secondary screening cascade intended to validate primary screening data and further explore the potential of a limited group of molecules to be tested in vivo. A set of 27 compounds was selected, representing 12 distinct chemical clusters. Following this rationale, hit compounds clearly fulfilled two key criteria in a process of drug candidate selection regarding necroptosis inhibition: (1) biochemical confirmation of ability to interfere with MLKL activation, the terminal mandatory component of the necroptosis pathway; (2) adequate safety-efficacy profile, crucial hurdle on early stage drug discovery and development^59^. Furthermore, the broad spectrum of action of tested compounds was confirmed at the end of the secondary phase using human intestine- and mice brain-derived cellular models. Seven of the 27 tested compounds completely preserved metabolic and morphological features of human HT29 and murine BV2 cells under necroptosis execution, avoiding bioenergetic breakdown of intracellular ATP levels and loss of membrane integrity. These results support the existence of molecular mechanisms of action involving conserved key players of necroptosis for these compounds, under different stimuli, cell-derived tissues and species.

Lastly, the results of the SIRS animal experiment demonstrated the ability of one of the seven screening hits, to protect in vivo from necroptosis execution. As direct hit from the screening cascade, proof of concept SN-6109 displayed high potency on mice and human cell models, no apoptosis modulation, RIPK1 kinase inhibitory activity, lack of toxicity and acceptable pharmacokinetic properties making it fit for further chemistry development and synthesis of new analogues.

In conclusion, here we describe a new cell-based phenotypic HTS workflow strategy for the discovery and selection of new necroptosis inhibitors. Laid on three distinct assay stages and ten individual steps using in vitro, *in silico* and in vivo methodologies, this screening workflow ensured a confident hit rate of active molecules with a low rate of false positives, thus overcoming some disadvantages and hurdles of phenotypic compound screening strategies. Moreover, new molecular scaffolds capable of interfering with necroptosis execution are currently undergoing medicinal chemistry development, with elucidation of MMoA, and further studies in disease-relevant pre-clinical models.

## Materials and methods

### Cell culture and reagents

Human liver HepG2 and murine fibroblast L929 cell lines were grown in DMEM medium. Human T lymphocyte Jurkat FADD^−/−^, Jurkat E6.1, murine microgial BV2 and human colon adenocarcinoma HT29 cell lines were grown in RPMI medium. Cell media were supplemented with 10% FBS, 1% antibiotic/antimycotic and other supplements according to each cell line specificities. All cell lines were cultured at 37 °C under a humidified atmosphere of 5% CO_2_.

### Chemical screening library

A library of 251,328 diverse small-molecule compounds (AstraZeneca) in 0.1% DMSO was arrayed in white 384-well microtiter plates at pre-established amounts or provided solubilized in DMSO at 10 mM. The compounds were selected as a representative subset of the entire corporate screening library to provide maximal chemical diversity, while retaining good physicochemical properties.

### Cell treatments and viability assays

For necroptosis induction, m/hTNF-α, Z-VAD-FMK, and/or Smac mimetic BV6 were used depending on each cell line. For apoptosis assays, Jurkat E6.1 cells were incubated with CHX. All cells were co-incubated with test compounds at target concentrations for 8 or 24 h. For cytotoxicity assays, cells were incubated with test compounds at target concentrations for 24 h. Viability assays were performed according to manufacturers’ instructions. Further details are provided as Supplementary Information.

### Primary high-throughput screen

L929 cells were co-incubated for 8 h with mTNF-α and test compounds at 31.7 μM after which AK release was measured. Compounds promoting necroptosis inhibition superior to 30% were considered as positive hits. Hit compounds half maximal effective concentration (EC_50_) was assessed through a 10-point dose-response curve in both L929 and Jurkat FADD^−/−^, and hit compounds were selected by a cut off piEC_50_ > 5 in both cell lines. Hit compounds were then evaluated for apoptosis modulation. Further details are provided as Supplementary Information.

### RIPK1 and RIPK3 kinase activity assays

Hit compounds were tested at 1 μM for both RIPK1 and RIPK3 kinase activity. RIPK1 kinase activity was evaluated by a radiometric-binding assay using myelin basic protein as substrate (Eurofins, France), while RIPK3 kinase activity was evaluated using a FRET-based assay (Thermo Fisher Scientific, Waltham, MA, USA). Further details are provided as Supplementary Information.

### Protein extraction and immunoblotting

Total proteins were extracted from both L929 cells and mouse liver tissue following standard protocols. Steady-state protein expression levels of MLKL, p-MLKL, RIPK3 and p-RIPK3 were determined by immunoblot analysis. β-actin was used as loading control. Further details are provided as Supplementary Information.

### Computational methods

Three-dimensional structures of human RIPK1 were selected and prepared using the Molecular Operating Environment software package. Co-crystallized ligands directly extracted from the corresponding X-ray structures were useful to define the protein binding site. To understand the activity of SN-6109 compound and Nec-1 against RIPK1, molecular docking calculations (non-covalent) were carried out using GOLD 5.7 program from CSD-Discovery Suite. Further details are provided as Supplementary Information.

### Animal studies, injections and monitoring

Male C57BL/6J mice were challenged with mTNF-α (9.5 µg) in the presence and absence of SN-6109 (125 µg). Nec-1 was positive control for TNF-induced SIRS protection. Body temperature and mortality were monitored up to 96 h. Further details are provided as Supplementary Information.

### Statistical analysis

Statistical analysis was performed using GraphPrism version 6.01 software (GraphPad Software, Inc., CA, USA). All data from the secondary screening cascade are expressed as mean ± SEM from at least three independent experiments. Statistical significances were determined using one or two-way analysis of variance (ANOVA) with Bonferroni post-hoc test. Body temperature is shown as mean ± SEM and compared with one-way analysis of variance (ANOVA) with Bonferroni post-test, while Kaplan–Meier survival curves were compared using log-rank (Mantel-Cox) test. *p*-value inferior to 0.05 was considered as statistically significant.

## Supplementary information

Supplemental text

Figure 1S

Figure 2S
