# Supplementary material for: Phenotypic high-throughput screening platform identifies novel chemotypes for necroptosis inhibition
Source: Cell Death Discov. 2020 Feb 11;6:6. doi: 10.1038/s41420-020-0240-0 (PMC7026080; doi:10.1038/s41420-020-0240-0)
Supplement: Supplementary file 1 — Supplemental text [file 41420_2020_240_MOESM1_ESM.docx]

Phenotypic high-throughput screening platform identifies novel chemotypes for necroptosis inhibition

Hugo Brito,^1^ Vanda Marques,^1^ Marta B. Afonso,^1^ Dean G. Brown,^2^ Ulf Börjesson,^3^ Nidhal Selmi,^3^ David M. Smith,^4^ Ieuan O. Roberts,^4^ Martina Fitzek,^5^ Natália Aniceto,^1^ Rita C. Guedes,^1^ Rui Moreira,^1^ Cecília M.P. Rodrigues*^1^

^1^Research Institute for Medicines (iMed.ULisboa), Faculty of Pharmacy, Universidade de Lisboa, 1649-003 Lisbon, Portugal; Hit Discovery, Discovery Sciences, R&D Biopharmaceuticals, AstraZeneca, ^2^Boston, MA 02451 USA, ^3^Gothenburg, 431 83 Sweden, ^4^Cambridge, CB4 0WG UK, ^5^Alderley Park, Macclesfield, SK10 4TG UK

Running title: Novel necroptosis inhibitors

*To whom correspondence should be addressed. E-mail: cmprodrigues@ff.ulisboa.pt

Keywords: HTS; necroptosis inhibitors; RIPK; MLKL; TNF-induced systemic inflammatory response syndrome

Detailed Supplementary Information

Materials and Methods

**Cell culture and Reagents.** L929 murine fibrosarcoma, FADD-deficient (FADD^-/-^) human Jurkat T-cell line, FADD-efficient human Jurkat E6.1 T-cell line, HT29 human colorectal adenocarcinoma cell line and BV2 murine microglia cells were originally obtained from ATCC Collections (Manassas, Virginia, USA). HepG2 hepatocellular carcinoma cells were from ECACC Culture Collection (Salisbury, UK). HepG2 and L929 cell lines were grown in Dulbecco’s modified Eagle’s medium, and Jurkat FADD^-/-^, Jurkat E6.1, BV2 and HT29 cell lines were grown in Roswell Park Memorial Institute 1640 Medium (Gibco, ThermoFisher Scientific, Paisley, UK). Media were supplemented with 10% heat-inactivated fetal bovine serum and 1% antibiotic/antimycotic solution (ThermoFisher Scientific). The medium for BV2 cells was additionally supplemented with 1% GlutaMAX^TM^ and the medium for HepG2 cells with 1% non-essential aminoacids (ThermoFisher Scientific). All cell lines were cultured at 37ºC under a humidified atmosphere of 5% CO_2_. Other reagents used were as follows: cycloheximide (CHX; Sigma-Aldrich, St. Louis, MO, USA), (2-Hydroxypropyl)-β-cyclodextrin (Cyclodextrin; Sigma-Aldrich), dimethyl sulfoxide (DMSO; Sigma-Aldrich), ethanol (EtOH) 96.9% (Honeywell, New Jersey, USA), Nec-1 (Sigma-Aldrich), phosphate-buffered saline 1x (PBS; ThermoFisher Scientific), polyethylene glycol 400 (PEG 400; Merck Millipore, Darmstadt, Germany)), recombinant murine TNF-α (mTNF-α; Peprotech, London, UK), recombinant human TNF-α (hTNF-α; Peprotech), Tween 80 (Sigma-Aldrich), Smac mimetic BV6 (ApexBio, Houston, TX, USA), Z-Val-Ala-Asp-fluoromethylketon (Z-VAD-FMK) pan-caspase inhibitor (Enzo Life Sciences, Farmingdale, NY, USA.

**Cell treatments and viability assays.** Cells were seeded in 384- or 96-well plates at 1000 or 8000 cells/well (in 12.5 μL or 100 μL final volume), respectively, using a Multidrop^TM^ Combi Reagent Dispenser (ThermoFisher Scientific). For inducing necroptosis, L929 and Jurkat FADD^-/-^ cells were incubated with 10 ng/mL mTNF-α and hTNF-α respectively; BV2 cells with 25 µM Z-VAD-FMK, and HT29 cells with 20 ng/mL hTNF-α, 250 nM Smac mimetic BV6 and 10 μM Z-VAD-FMK. Cells were co-incubated in the presence of test compounds at target concentrations for 8 or 24 h. Nec-1 at 29.2 μM was used as positive control. For apoptosis assays, Jurkat E6.1 cells were co-incubated with 0.5 μg/mL CHX and test compounds at target concentrations for 8 h. In cytotoxicity assays, HepG2 and L929 cells were seeded in 96-well plates at 5000 cells/well, in 100 μL final volume. Twenty-four hours after seeding, cells were incubated with test compounds at 50 and 100 μM for additional 24 h.

Adenylate kinase (AK) and lactate dehydrogenase (LDH) release from damaged cells were measured using the Toxilight BioAssay Kit (Lonza, Basel, Switzerland) and the Cytotoxicity Detection Kit (Roche Diagnostics GmbH, Mannheim, Germany), respectively. Adenosine triphosphate (ATP) metabolism was evaluated using CellTiter-Glo^TM^ Luminescent Cell Viability Assay (Promega, Madison, WI, USA). Apoptosis was determined by the measurement of caspase-3/-7 activity using the Caspase-Glo^®^ 3/7 Assay (Promega) according to the manufacturer’s instructions. Cellular metabolic activity was measured using CellTiter 96^®^ Aqueous Non-Radioactive Cell Proliferation (MTS) Assay (Promega). Luminescence signal was recorded using a GloMax^®^-MultiDetection System (Promega) and absorbance with a Model 680 microplate reader (Bio-Rad Laboratories Inc, Hercules, CA, USA).

**Primary high-throughput screen.** Test compounds were arrayed in 384-well white microtiter plates and screened for their potential as necroptosis inhibitors at 32 μM target concentration. Eight hours after co-incubation of L929 cells with mTNF-α and test compounds, AK release was evaluated. For data normalization purposes, wells containing untreated cells or cells treated with TNF-α were included. L929 cells co-incubated with mTNF-α and SN-2668/Nec-1 were used as positive controls. For intraplate quality control purposes, a Z’ factor > 0.5 was considered of very good quality. Data was normalized to treated cell wells. Compounds with cell death inhibition of -30% (necroptosis inhibition superior of 30%) and with a Z Score of -10 were considered as positive hits. Data analysis was performed using GeneScreener^®^ Software (GENEDATA AG, Basel, Switzerland). Selected hits were then tested for effectiveness as necroptosis inhibitors by assessing their half maximal effective concentration (EC_50_) through a 10-point dose-response curve. Test compounds were arrayed in 384-well white microtiter plates, dispensed in a snake-like pattern at the point concentrations of 100, 32, 10, 4, 1, 0.4, 0.1, 0.04, 0.001 and 0.004 μM. Eight hours after co-incubation of L929 or Jurkat FADD^-/-^ cells with TNF-α and test compounds, AK release was evaluated. Drug potency (piEC_50_/ EC_50_) was extrapolated from the dose-response curve fit and hit compounds were selected by a cut off piEC_50_ > 5 (or EC_50_ < 6.7 μM) in both cell line assays. For evaluation of apoptosis modulation, hit compounds were arrayed in 384-well white plates as described before, at target concentrations of 0.03, 0.3, 3, 30 μM. Eight hours after co-incubation of Jurkat E6.1 cells with CHX and test compounds, caspase-3 and -7 activity was evaluated. Compounds were classified as inactive, active or weakly active in modulating apoptosis. Only inactive compounds were selected as positive hits.

**Protein extraction and immunoblotting.** L929 cells were seeded at 1.5x10^5^ cells/mL in 6-well plates. Twenty-four hours after seeding, cells were co-incubated with 10 ng/mL mTNF-α and test compounds at target concentrations. After 8 h of incubation, samples were recovered and homogenized in ice cold 1:1 solution of buffer A (10 mM Tris•HCl pH 7.6, 5 mM MgCl_2_, 1.5 mM KOAc, 2 mM dithiothreitol (DTT), and Halt Protease and Phosphatase inhibitor cocktail, EDTA-free; ThermoFisher Scientific) and buffer 2X (10 mM Tris•HCl pH 7.6, 1% Nonidet-P40, and Halt Protease and Phosphatase inhibitor cocktail) for 30 min. Protein lysates were sonicated and centrifuged at 10.000 g for 10 min at 4º C. Supernatants containing the total protein extracts were transferred to a fresh tube and total protein concentrations were determined using a NanoDrop ND-1000 Spectrometer (ThermoFisher Scientific). For total protein extraction, liver tissue was firstly homogenized using a glass dounce homogenizer in ice-cold lysis buffer. The remaining steps follow the protocol above described. Steady-state protein expression levels were determined by immunoblot analysis. Briefly, 50 μg of total protein extracts were denaturated, separated on 10% sodium dodecyl sulphatepolyacrylamide electrophoresis gels, and transferred onto nitrocellulose membranes. After blocking with 5% milk solution, blots were incubated overnight at 4ºC with primary rabbit antibodies reactive to p-MLKL (L929 cell line: 1:1000, AB196436; mouse tissue: 1:5000; AB187091; both from Abcam, Cambridge, UK), MLKL (L929 cell line: 1:500; mouse tissue: 1:5000; SAB1302339; Sigma-Aldrich), p-RIPK3 (1:5000; AB195117, Abcam) and RIPK3 (1:5000; SC-374639, Santa Cruz Biotechnology, CA, USA), and with a secondary antibody conjugated with horseradish peroxidase (Bio-Rad) diluted 1:3000 in blocking solution for 1 h at room temperature. β-actin (1:5000, A5541; Sigma-Aldrich) was used as loading control. Membranes were processed for protein detection by chemiluminescence using SuperSignal^TM^ West Fento Maximum Sensitivity Substrate (ThermoFisher Scientific) or Immobilon Western Chemiluminescent HRP Substrate (Millipore, Burlington, MA, USA), and acquired using the ChemiDoc XRS-imaging system (Bio-Rad). Data was analysed using ImageLab^TM^ software version 5.1 Beta (Bio-Rad).

**Computational methods.** The three-dimensional (3D) structures of human RIPK1 to be used in the docking studies were obtained from the Protein Data Bank (PDB). At the time of this study, there were seven different RIPK1 X-ray structures PDBID’s 4ITI, 4ITH, 4ITJ, 4NEU, 5HX6, 6C3E, and 6C4D (resolution between 1.80 - 2.89 Å) co-crystallized with known inhibitors. To prepare the proteins for the molecular docking studies, the co-crystallized inhibitors as well as all the crystallographic waters included in the PDB structures were removed. Hydrogen atoms were then added and the protonation and tautomeric states (specially Asp, Glu, Arg, Lys and His) were correctly assigned using the Protonate-3D tool within the Molecular Operating Environment (MOE 2019.01) software package at pH 7.4. Co-crystallized inhibitors were useful to define the protein-binding site. For ligand preparation, the seven co-crystallized ligands were directly extracted from the corresponding X-ray structures. Nec-1 and SN-6109 were built and energy minimized using Amber force field implemented in MOE 2019.01 software. Possible protonation and ionization states were assigned to each ligand at pH 7.4. Finally, to understand the activity of the studied compounds against RIPK1, molecular docking calculations (non-covalent) were carried out using GOLD 5.7 program from CSD-Discovery Suite (CCDC). No constraints were used in any calculation. Flexible ligand sampling was considered in the docking procedure. All other parameters were set to defaults for the GOLD docking process. Molecular docking studies were then performed using the all four scoring function from GOLD 5.7 software package and each ligand was subjected to 1,000 docking runs. The docking protocol was validated for all the seven structures prepared re-docking the crystallographic ligands and their poses were reproducible with RMSD´s below 1.5 Å. In addition, extra validation of RIPK1 protein structure was performed by cross-docking all ligands in each X-ray structure. Significant differences were observed between the final poses and scores obtained using these 7 different structures. Our calculations revealed that using ChemPLP as scoring function, 4ITH, 4ITJ and 6C3E were only able to suitably accommodate small ligands, but induced strong torsions on geometries of larger compounds. By contrary, X-ray structures such as 4ITI, 4NEU and, 6C4D preferentially accommodated large ligands. 5HX6 was the only X-ray structure that accommodated both large and small ligands and for this reason further used in this study.

**Pharmacokinetic measurements.** Pharmacokinetic studies were performed in male C57BL/6J mice by Pharmaron (Pharmaron Beijing Co Ltd, Beijing, China).  Compound (5 mg/kg body weight) was administered intravenously in vehicle (57% PBS, 32% DMSO, 4% PEG 400, 3% ethanol, 4% Tween 80). Blood samples obtained postdosing were collected in plastic microcentrifuge tubes containing EDTA-K2 and centrifuged at 4000 × g for 5 min to obtain plasma. The concentration of compound in plasma samples was determined using a liquid chromatography-tandem mass spectrometry (LC-MS/MS) method by monitoring transition from m/z 541 to 383 in ESI-positive mode. PK parameters were calculated by non-compartmental model using Phoenix WinNonlin v. 6.1 (Pharsight Corp, Certara, St. Louis, MO, USA).

**Animal studies, injections and monitoring.** Male C57BL/6J mice with 18-26 g (Charles River Laboratories International, Inc., Wilmington, MA, USA) were used at the age of 8-14 weeks. Five to seven animals were included in each experimental group. All animal experiments were carried out with the permission of local animal ethical committee in accordance with the EU Directive (2010/63/EU), Portuguese laws (DL113/2013, 2880/2015, 260/2016) and all relevant legislations. Animals received humane care in a temperature-controlled environment with a 12 h light-dark cycle and *ad libitum* access to pelleted chow and water, complying with the Institute’s guidelines, and as outlined in the “Guide for the Care and Use of Laboratory Animals” prepared by the National Academy of Sciences and published by the National Institutes of Health (NIH publication 86-23 revised 1985). To validate the anti-necroptotic potential of a test compound on TNF-induced systemic inflammatory response syndrome (SIRS), mice were challenged with mTNF-α in the presence and absence of SN-6109, while Nec-1 posed as positive control for TNF-induced SIRS protection. mTNF-α was diluted in sterile ultrapure Type 1 water. Nec-1 was diluted as follows (v/v): 48% PBS 1X, 17% DMSO and 35% PEG 400. SN-6109 was diluted (v/v) in 57% PBS 1x, 32% DMSO, 4% PEG 400, 3% ethanol, 4% Tween 80 and 2% Cyclodextrin. Vehicle control, SN-6109 or Nec-1 (125 µg; 5 mg/kg body weight) were injected 15 min before mTNF-α challenge (9.5 µg; 0.38 mg/kg body weight). All injections were administered intravenously (i.v.) in 100 µL volume each. Body temperature was recorded on mice pre-shaven back using a non-contact infrared thermometer suitable for animal use (BIO-IRB153; BioSeb, Vitrolles, France). Body temperature and mortality were monitored up to 96 h. Animals were euthanized by inhalative induction of narcosis with isoflurane at the end of the experiment, or at humane endpoints according to the Clinical Severity Score ^59^.

**FIGURE LEGENDS**

**Fig. S1**. Detailed assay workflow for the discovery of new necroptosis inhibitors through the screening of a 251,328 compound library. Schematic overview of the complete compound screening strategy (left). Detailed overview of the methodology and hit selection criteria for the automated phenotypic HTS steps from workflow stage I (right).

Fig. S2. Preliminary studies for the optimization and validation of *in vitro* screening assays. (A) Evaluation of Nec-1 ability to prevent necroptosis execution in murine L929 and human Jurkat FADD^-/-^ cells exposed to 10 ng/mL mTNF-α and hTNF-α, respectively, for 8 h. Apoptosis modulation by Nec-1 and Z-VAD-FMK was evaluated in human Jurkat E6.1 cells incubated with 0.5 μg/mL CHX, 29.2 μM Nec-1 or 10 μM Z-VAD-FMK for 8 h. Cell viability was assessed using a luminescence-based readout for AK release and Caspase-Glo 3/7 throughout. (B) Modulation of p-MLKL/MLKL protein ratio was evaluated in murine L929 cells exposed to 10 ng/mL mTNF-α and 29.2 μM Nec-1 for 8 and 24 h. Whole cell extracts were subjected to immunoblotting using the indicated antibodies. Data represent mean values ± SEM of three independent experiments normalized to untreated control. ***p* < 0.01 and ****p* < 0.001.
