# Supplementary figures and images for: Phenotypic high-throughput screening platform identifies novel chemotypes for necroptosis inhibition
Source: Cell Death Discov. 2020 Feb 11;6:6. doi: 10.1038/s41420-020-0240-0 (PMC7026080; doi:10.1038/s41420-020-0240-0)

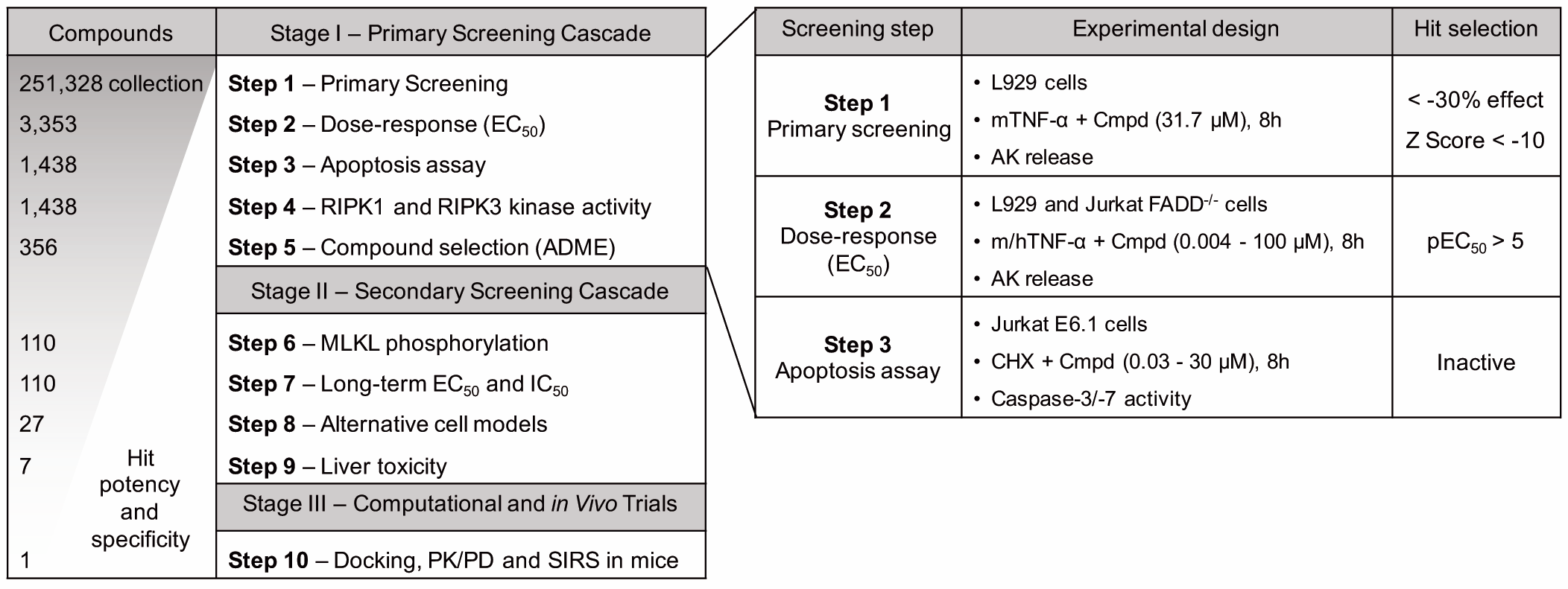

Supplement: Supplementary file 2 — Figure 1S [file 41420_2020_240_MOESM2_ESM.tif]

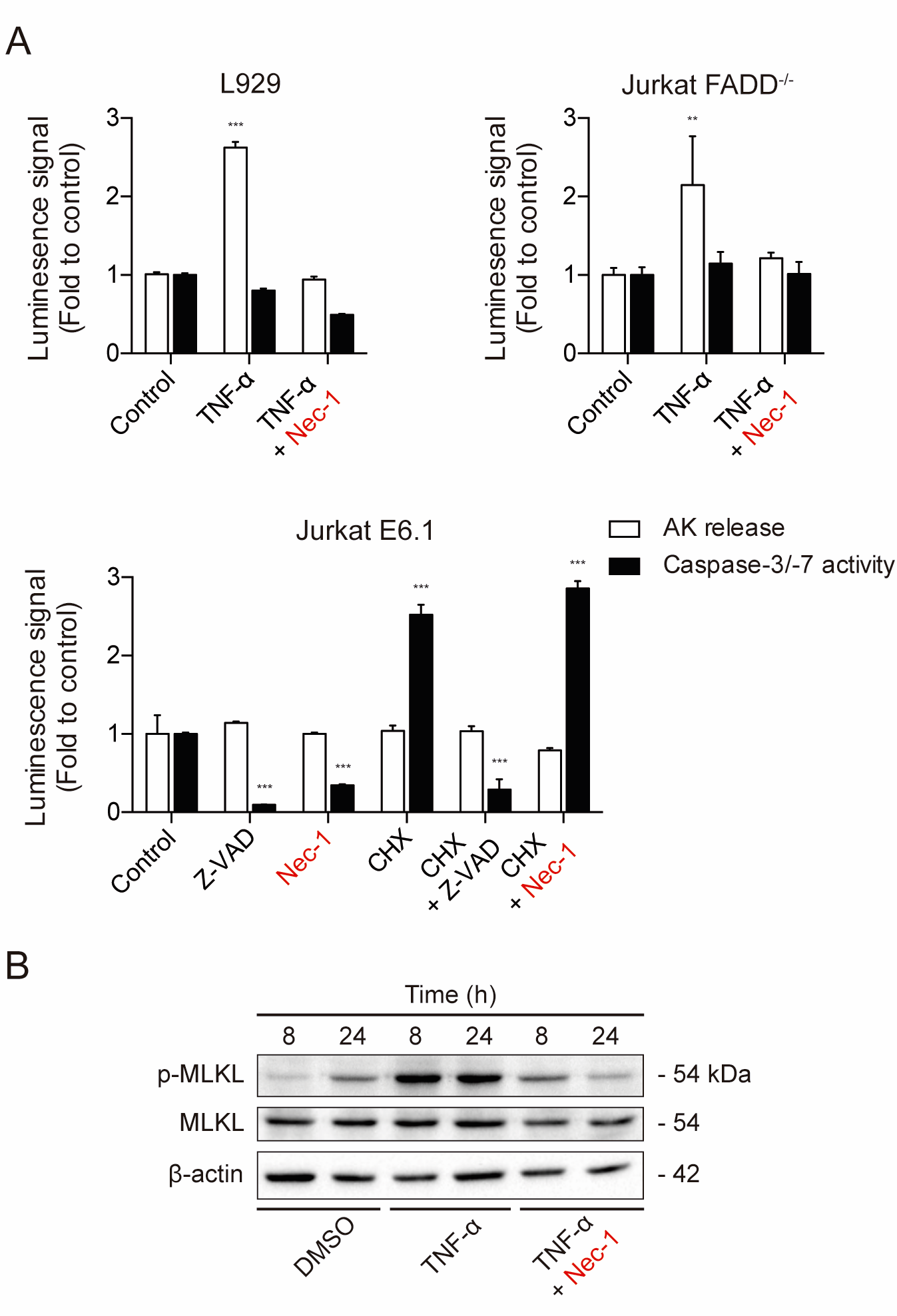

Supplement: Supplementary file 3 — Figure 2S [file 41420_2020_240_MOESM3_ESM.tif]
